# Impact of systematic diabetes screening on peri-operative infections in patients undergoing cardiac surgery

**DOI:** 10.1038/s41598-024-65064-7

**Published:** 2024-06-20

**Authors:** Alessandro Mattina, Giuseppe Maria Raffa, Maria Ausilia Giusti, Elena Conoscenti, Marco Morsolini, Alessandra Mularoni, Maria Luisa Fazzina, Daniele Di Carlo, Manlio Cipriani, Francesco Musumeci, Antonio Arcadipane, Michele Pilato, Pier Giulio Conaldi, Diego Bellavia

**Affiliations:** 1 Diabetes Service, IRCCS-ISMETT (Istituto Mediterraneo per i Trapianti e Terapie ad alta specializzazione), UPMC (University of Pittsburgh Medical Center), 90127 Palermo, Italy; 2 Heart Center, IRCCS-ISMETT (Istituto Mediterraneo per i Trapianti e Terapie ad alta specializzazione), UPMC (University of Pittsburgh Medical Center), 90127 Palermo, Italy; 3 Directorate of Health Professions, IRCCS-ISMETT (Istituto Mediterraneo per i Trapianti e Terapie ad alta specializzazione), UPMC (University of Pittsburgh Medical Center), 90127 Palermo, Italy; 4 Unit of Infectious Diseases and Infection Control, IRCCS-ISMETT (Istituto Mediterraneo per i Trapianti e Terapie ad alta specializzazione), UPMC (University of Pittsburgh Medical Center), 90127 Palermo, Italy; 5 Quality and Accreditation Department, IRCCS-ISMETT (Istituto Mediterraneo per i Trapianti e Terapie ad alta specializzazione), UPMC (University of Pittsburgh Medical Center), 90127 Palermo, Italy; 6 Laboratory of Clinical Pathology, Microbiology and Virology, IRCCS-ISMETT (Istituto Mediterraneo per i Trapianti e Terapie ad alta specializzazione), UPMC (University of Pittsburgh Medical Center), 90127 Palermo, Italy; 7 Department of Anesthesia and Intensive Care, IRCCS-ISMETT (Istituto Mediterraneo per i Trapianti e Terapie ad alta specializzazione), UPMC (University of Pittsburgh Medical Center), 90127 Palermo, Italy; 8 Department of Research, IRCCS-ISMETT (Istituto Mediterraneo per i Trapianti e Terapie ad alta specializzazione), UPMC (University of Pittsburgh Medical Center), 90127 Palermo, Italy

**Keywords:** Endocrinology, Health care, Medical research, Infectious diseases, Metabolic disorders

## Abstract

Detection of high glycated hemoglobin (A1c) is associated with worse postoperative outcomes, including predisposition to develop systemic and local infectious events. Diabetes and infectious Outcomes in Cardiac Surgery (DOCS) study is a retrospective case–control study aimed to assess in DM and non-DM cardiac surgery patients if a new screening and management model, consisting of systematic A1c evaluation followed by a specialized DM consult, could reduce perioperative infections and 30-days mortality. Effective July 2021, all patients admitted to the cardiac surgery of IRCCS ISMETT were tested for A1c. According to the new protocol, glucose values of patients with A1c ≥ 6% or with known diabetes were monitored. The diabetes team was activated to manage therapy daily until discharge or provide indications for the diagnostic-therapeutic process. Propensity score was used to match 573 patients managed according to the new protocol (the Screen+ Group) to 573 patients admitted before July 2021 and subjected to the traditional management (Screen−). Perioperative prevalence of infections from any cause, including surgical wound infections (SWI), was significantly lower in the Screen+ as compared with the Screen− matched patients (66 [11%] vs. 103 [18%] *p* = 0.003). No significant difference was observed in 30-day mortality. A1c analysis identified undiagnosed DM in 12% of patients without known metabolic conditions. In a population of patients undergoing cardiac surgery, systematic A1c evaluation at admission followed by specialist DM management reduces perioperative infectious complications, including SWI. Furthermore, A1c screening for patients undergoing cardiac surgery unmasks unknown DM and enhances risk stratification.

## Introduction

Diabetes mellitus (DM) is an extremely common comorbidity involving approximately 10% of patients undergoing surgery and 25% of patients undergoing coronary artery revascularization^1^. Morbidity and mortality rates of such patients, specifically in the cardiac surgical setting, are around 30% higher compared with patients without diabetes, partly due to the increased risk of infection^2–4^. Hyperglycemia, whether from surgical stress or related to underlying disease associated with known or unknown diabetes, has been systematically identified as a marker of worse prognosis in hospitalized patients in any care and surgical setting^5,6^. Moreover, detection of high glycated hemoglobin (A1c) is associated with worse postoperative outcomes^7,8^. Indeed, the presence of DM further prolongs hospitalization, increases the risk of complications and the predisposition to developing both systemic and local infectious events, and incurs significant increases in healthcare costs^9^.

Several metabolic factors, including obesity, malnutrition, dyslipidemia, and hyperglycemia, have been identified as predictors of the risk of postoperative infection^10^. DM and acute hyperglycemia are associated with chronic inflammation and can increase the risk of infection by impairing immune function and promoting bacterial growth^11,12^. DM is a well-established risk factor for surgical wound infections (SWI), and A1c has been reported as an independent predictor of SWI^13^.

Accurate glycemic control, resulting from comprehensive screening, reduces infectious episodes and total mortality in patients referred for cardiac surgery^8,14^. Therefore, our hypothesis was that systematic screening of patients referred to cardiac surgery through A1c measurement allows the unmasking of the presence of undiagnosed diabetes, assessment of glycemic control, and risk stratification of peri-operative infections, also providing the opportunity to reduce such infections. Furthermore, the A1c level measured at admission has been demonstrated to be associated with glycemic control during hospitalization and can thereby impact therapeutic decisions in a standardized specialist consult setting^15,16^.

The aim of the DOCS study (Diabetes and infectious Outcomes in Cardiac Surgery) was to test whether systematic screening of A1c levels, coupled with subsequent specialistic management when needed, reduced perioperative outcomes in patients undergoing cardiac surgery in terms of total infectious episodes (including SWI) as well as mortality for any cause, within 30 days from discharge.

## Research design and methods

### Study population and design

**Figure 1:**
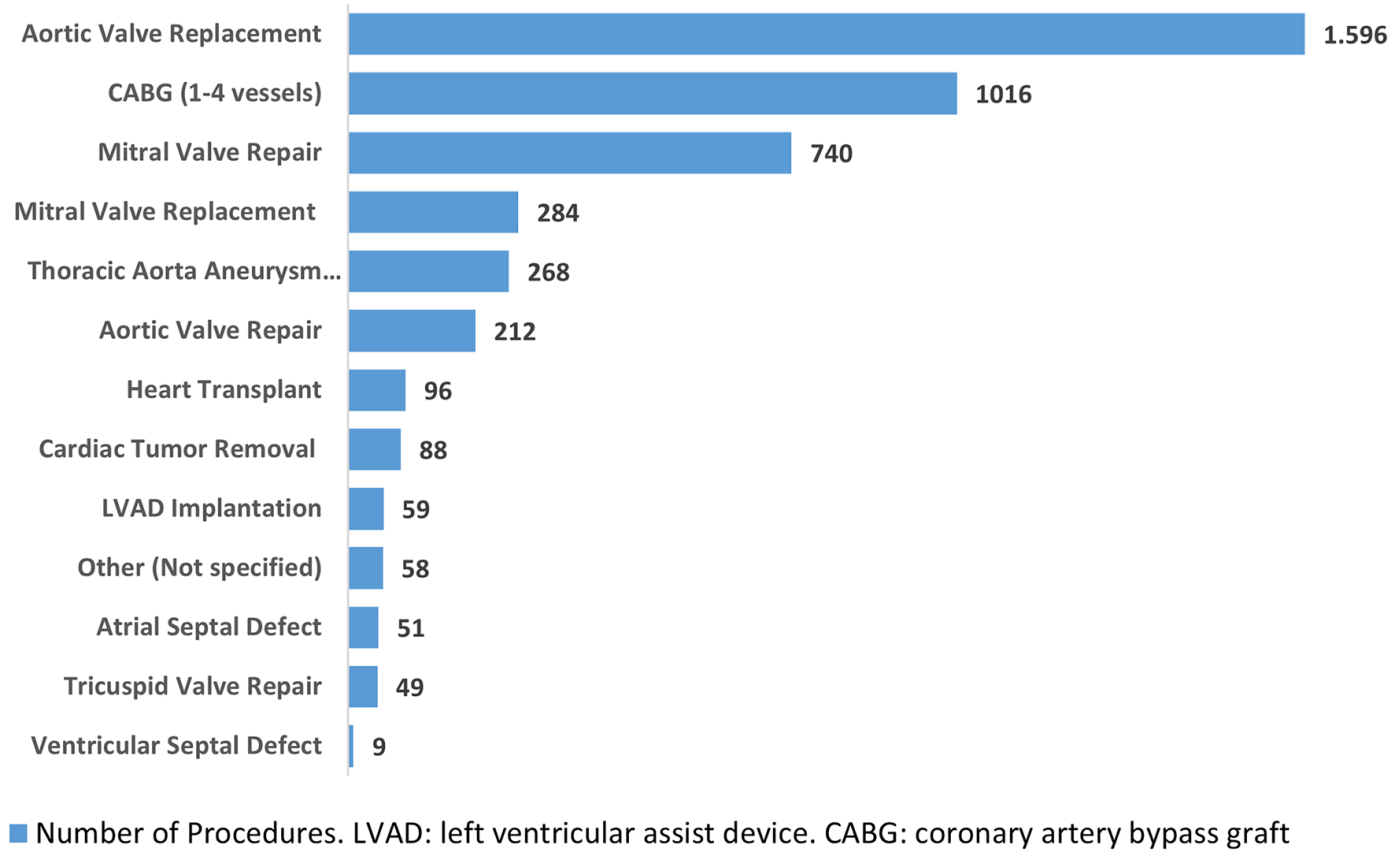
Surgery procedures performed.

Patients admitted to the cardiothoracic surgery department at UPMC | IRCCS ISMETT (Palermo, Italy) and referred for cardiac surgery from June 1st, 2014, through September 30th, 2022, (N = 4526) were eligible for recruitment. The surgical procedures included valve surgery, coronary artery bypass graft (CABG), heart transplantation, left ventricular assist device (LVAD) implantation, and other aortic surgeries (Fig. 1). Patients referred for percutaneous approaches were excluded.

A subset of 597 patients, all admitted after June 2021, underwent systematic screening for diabetes as described above. Diabetes specialist consult consisted of following the trend of glycemic values and modulating the therapy daily until the values stabilized for more than 3 consecutive days or until discharge of the patient.

Diabetes was managed according to the American Diabetes Association recommendations^17^. Point-of-care glucose monitoring (at four points: before meals and two hours after dinner) was performed in all patients with diabetes or with A1c ≥ 6% (42 mmol/mol). In diabetic patients referred for surgery, any therapy with oral or subcutaneous non-insulin hypoglycemic drugs was suspended per protocol. All patients with glycemia ≥ 180 mg/dl in one or more measurements, both with known diabetes and with diabetes and/or hyperglycemia detected on admission, were referred to diabetes counseling and basal insulin therapy or a basal bolus regimen was implemented.

**Figure 2:**
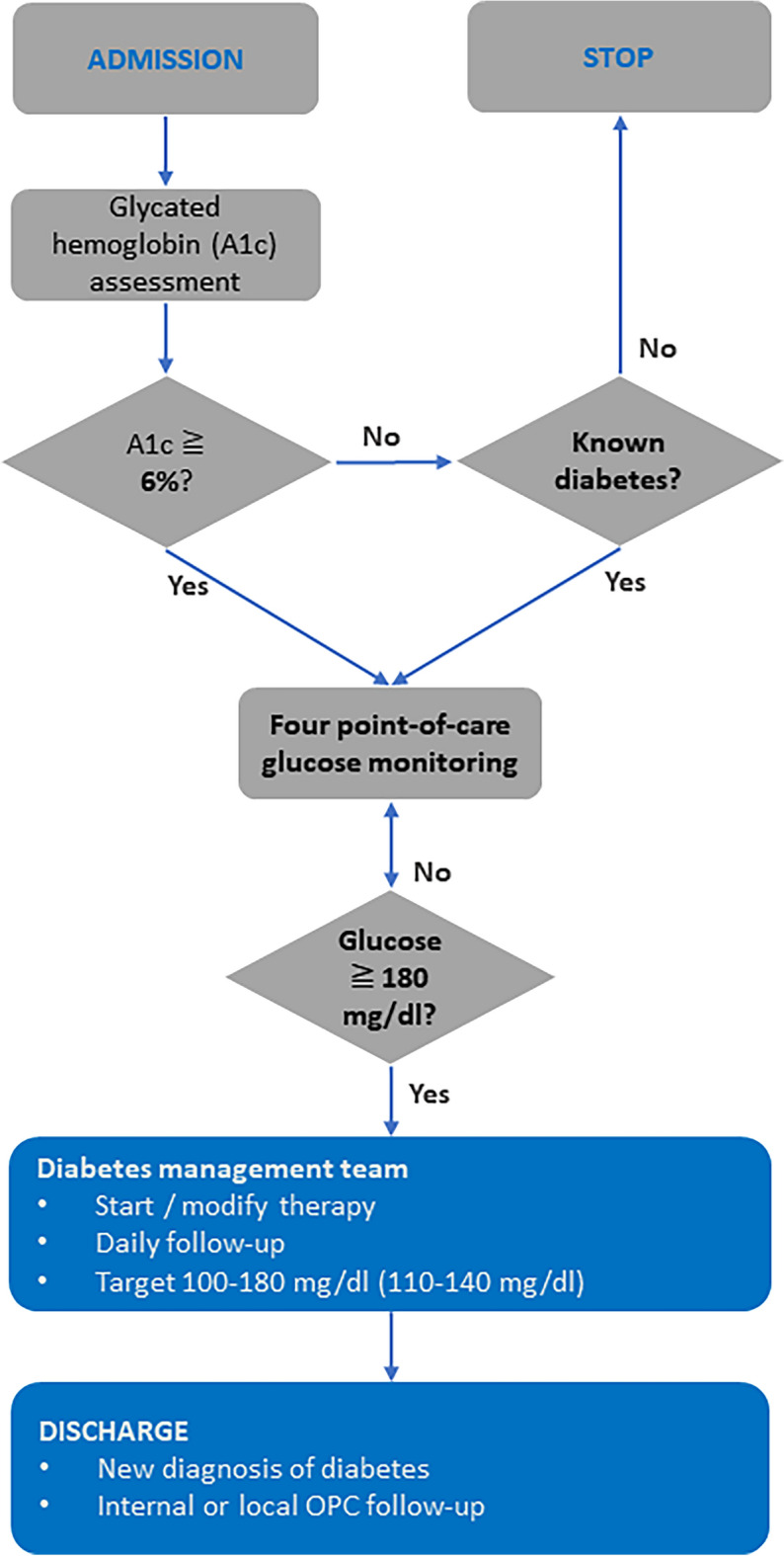
Cardiac surgery patient pathway for assessment of glucometabolic compensation and diabetes management.

The therapeutic target glycemic range in the perioperative phase was 100–180 mg/dl. This range considers the need to find an appropriate balance between achieving good glycemic control and avoiding hypoglycemic episodes, which are evidently associated with an increased risk of mortality^18^. However, given the data in the literature on the greater benefit of a more stringent target range in specific cardiac surgery patients, a target of 110–140 in the early postoperative period was considered when hypoglycemic risk was low^19^. Day-to-day treatment decisions were made by the diabetes team, involving dietitians and specialist nurses, taking into consideration associated diseases and therapy. Upon discharge, patients were referred to the local diabetology service with a defined plan of care (Fig. 2).

Demographic, biometric, and clinical variables were collected, as well as routine laboratory values, including measures of inflammation and metabolic balance. In addition, incidence of infection (in any location) was collected from the hospital's internal surveillance database and internal data from the Society of Thoracic Surgeons (STS) Database, an international observational register approved by the ethics committee (IRB 57/17) and used to record data from our cardio-surgical population since 2014. Definitions for all the studied variables are cited from the STS manual unless otherwise defined (Supplement).

From this population, two groups, each consisting of 573 1:1-propensity score matched patients (see “Statistical analysis”), were created:

1. *Screen− (negative)* group: patients admitted to UPMC | IRCCS ISMETT from June 1st, 2014, through June 31st, 2021, who did not receive systematic screening for unknown/uncontrolled diabetes (i.e., A1c measure). Specialist diabetes consult was requested only in the case of persistent hyperglycemia in patients with a history of diabetes.
2. *Screen*+ *(positive)* group: patients admitted from July 1st, 2021, through September 30th, 2022, referred for cardiac surgery, who did undergo systematic screening for unknown/uncontrolled diabetes (i.e., A1c measure), followed by specialist diabetes consult in case of A1c ≥ 42 mmol/mol (6%), regardless of diabetes status.

Informed consent was obtained from the patients. This retrospective study was conducted in compliance with the Declaration of Helsinki and approved by the local ethics committee (ISMETT IRRB/09/21-Sept. 2022).

The primary endpoint was postoperative short-term (i.e., 30 days) infection at any location. The secondary endpoint was 30-day all-cause mortality.

### Infection prevention and control

Every day, infection control nurses systematically review medical records to identify clinical data associated with infection events. Then, based on CDC/NHSN criteria, infectious disease specialists discuss and validate infection cases during a weekly multidisciplinary meeting.

In our hospital, a comprehensive active surveillance system is implemented to monitor and track hospital-acquired infections, specifically focusing on pulmonary infections related to mechanical ventilation, urinary tract infections associated with bladder catheters, bacteremia linked to intravascular devices, and surgical site infections across various surgical specialties such as abdominal, cardiac, and thoracic surgery^20,21^.

Specific indicators for infections caused by alert microorganisms, such as methicillin-resistant Staphylococcus aureus (MRSA), vancomycin-resistant Enterococci (VRE), carbapenem-resistant Enterobacteriaceae (CRE), Pseudomonas aeruginosa, multidrug-resistant (MDR) Acinetobacter baumannii, and extended-spectrum beta-lactamase (ESBL)-producing Enterobacteriaceae are monitored.

As part of our screening for multidrug-resistant organisms (MDRO), a rectal swab is performed at admission and repeated weekly. Nasal screening for Staphylococcus aureus is also performed at admission on every patient. If positive, a 5-day decolonization therapy regimen with daily antiseptic chlorhexidine showers and nasal mupirocin is prescribed.

Cefazolin antibiotic prophylaxis is administered 60 min before the scheduled time of surgery to all patients. A second dose of cefazolin is administered if the following conditions are met: (a) the surgery lasts longer than four hours; (b) there is blood loss greater than 1500 mL; or (c) the surgical field becomes accidentally contaminated.

Infection prevention complies with all international recommendations. This entails shaving practice prior to surgery, skin preparation, wound care, and glycemic control, as well as monthly air and surface surveillance to look for potential contamination in the operating room.

### Statistical analysis

Data are reported as mean ± SD for continuous parametric variables, median (interquartile range) for continuous nonparametric variables, and as frequency (percentage) for categorical variables.

To prevent potential selection bias in testing clinical usefulness of diabetic screening strategy, a stepwise strategy was adopted: first, a propensity score was computed, reflecting the probability that a patient would undergo screening for diabetes^22^. This was accomplished by performing a multivariable logistic regression analysis using screening as the dependent outcome variable and entering all demographics, history, echocardiography, and laboratory measurements, as well as surgical characteristics that would likely affect the probability of undergoing diabetes screening (Tables S1, S2). These variables included age, gender, primary clinical problem for which cardiac surgery was performed and whether it was an urgent procedure and/or a reoperation, body mass index, NYHA class, history of smoking, hypertension, COPD, chronic hemodialysis, coronary artery disease, liver disease, peripheral artery disease, cerebrovascular accident, coronary artery bypass graft, previous acute myocardial infarction, previous acute heart failure, preoperative need for treatment with cefazolin and/or mupirocin, MELD score, STS score for postoperative mortality, preoperative levels of C-reactive protein (CRP), white blood cells (WBC), hemoglobin, serum creatinine, left ventricular ejection fraction by ECHO, need for intra-aortic balloon pump preoperatively or intraoperatively, and need for intraoperative packed red blood cells transfusions.

Second, the derived propensity score was used to match the 595 patients in the Screen+ group to the 3929 patients in the Screen− group, using a 1:1 nearest neighbor matching algorithm with a caliper of 0.10. Implementing this algorithm, 22 patients from the Screen+ group could not be matched to any patient from the Screen− group, and the final analysis was performed on 573 matched pairs of Screen+ / Screen− patients.

After propensity score matching, covariate balance was assessed using absolute standardized mean differences (SMDs) between Screen+ and Screen− patients (Tables S1, S2; Supplement). Once matching by SMD was considered appropriate, two-sided t-tests or Mann–Whitney rank-sum tests were used to compare continuous variables according to normal/non-normal distribution assumption, while Fisher’s exact test was used for categorical variables.

A difference was considered statistically significant at α level of 0.05. Statistical analyses were performed using R version 4.22 (R Foundation for Statistical Computing, Vienna, Austria) and Stata version 17.1 (Stata-Corp LP, College Station, TX) software packages.

### Ethical approval

Only data from patients who consented to the processing of their personal data were collected. This retrospective study was conducted in compliance with the Declaration of Helsinki and approved by the local ethics committee (ISMETT IRRB/09/21-Sept. 2022).

## Results

The mean age of the whole cohort (N = 1146) was 65 years, with a higher number of male patients and a mean body mass index (BMI) in the overweight range (27.7 kg/m^2^). Active cigarette smokers were 17%. About a quarter of the patients had known diabetes, and 80% suffered from arterial hypertension. Approximately 4% had a history of cancer, 3% severe liver disease, and 2% end-stage renal failure in replacement therapy.

The most common causes of cardiac surgery admissions were aortic or mitral valve disease, coronary atherosclerosis needing coronary artery bypass graft (CABG), thoracic aneurysm, and acute and subacute forms of ischemic heart disease (Fig. 1). 93% of patients were undergoing their first cardiac surgery, and most admissions were elective (79%) or urgent (21%). Approximately 10% of patients had a history of acute myocardial infarction or stroke, and 5% had acute heart failure. Approximately 10% of patients had a history of percutaneous coronary intervention (PCI) and drug-eluting stent (DES) implantations. The percentage of patients with NYHA III/IV, suggestive of moderate/severe chronic heart failure, was 23%. The patients also showed a prevalence of chronic obstructive pulmonary disease of approximately 15%, especially of moderate grade.

All the previous parameters described, as well as the main preoperative laboratory and procedure parameters and prophylactic therapies, were considered in the propensity score matching and were, therefore, evenly distributed in the two study groups (Table S2).

In the comparative analysis, there were no significant differences between the Screen− (N = 573) and Screen+ (N = 573) groups in terms of age, sex, BMI, smoking status, cardiovascular risk factors, diabetes, or other pre-existing medical conditions (including liver diseases, cancer, coronary artery disease, stroke, heart failure or arrhythmias) (Table 1).

**Table 1:** Whole population characteristics and comparison between Screen+ and Screen− groups.

	All	Screen−	Screen+	*p*-value
n	1146	573	573	
*Characteristics and medical history*				
Age, years (mean ± SD)	64.7 ± 11.6	64.7 ± 11.6	64.8 ± 11.6	0.9
Females, n (%)	377 (32.9)	187 (32.6)	190 (33.2)	0.9
Height, cm (mean ± SD)	166 ± 9.5	166 ± 9.5	166 ± 9.5	0.975
Weight, Kg (mean ± SD)	75.4 ± 15.3	75.4 ± 15.3	75.4 ± 15.3	0.971
BMI, kg/m^2^ (mean ± SD)	27.3 ± 4.6	27.3 ± 4.7	27.3 ± 4.6	0.933
Smoking, n (%)	195 (17)	93 (16.2)	102 (17.8)	0.529
Hemodialysis, n (%)	21 (1.8)	11 (1.9)	10 (1.7)	1
Liver disease, n (%)	33 (2.9)	17 (3.0)	16 (2.8)	1
Diabetes mellitus, n (%)	279 (24.4)	144 (25.1)	135 (23.6)	0.582
History of infective endocarditis, n (%)	38 (3.3)	22 (3.8)	16 (2.8)	0.409
History of cancer, n (%)	50 (4.4)	24 (4.2)	26 (4.5)	0.885
*Cardiovascular history*				
Arterial hypertension, n (%)	916 (80.0)	456 (79.6)	460 (80.3)	0.825
Coronary artery disease, n (%)	74 (6.5)	32 (5.6)	42 (7.3)	0.279
Peripheral arterial disease, n (%)	47 (4.1)	19 (3.3)	28 (4.9)	0.233
Carotid artery stenting, n (%)	60 (5.3)	29 (5.1)	31 (5.4)	0.894
Cerebrovascular accident, n (%)	112 (9.8)	55 (9.6)	57 (9.9)	0.921
Acute myocardial infarction, n (%)	131 (11.5)	63 (11.0)	68 (11.9)	0.71
Acute heart failure, n (%)	55 (4.8)	25 (4.4)	30 (5.2)	0.58
NYHA Class III/IV, n (%)	257 (23)	122 (11)	135 (12)	0.93
Arrhythmia, n (%)	253 (22.1)	140 (24.4)	113 (19.7)	0.064
Pacemaker implantation, n (%)	11 (1.0)	4 (0.7)	7 (1.2)	0.545
Left ventricular assist device, n (%)	7 (0.6)	5 (0.9)	2 (0.3)	0.448
History of PCI and DES implantation, n (%)	109 (9.5)	50 (8.7)	59 (10.3)	0.421
*STS predicted risk scores*				
Morbidity or mortality (mean ± SD)	0.12 ± 0.09	0.12 ± 0.08	0.13 ± 0.10	0.348
Deep sternal wound infection (mean ± SD)	0.00 ± 0.00	0.00 ± 0.00	0.00 ± 0.00	0.655
Renal failure (mean ± SD)	0.03 ± 0.04	0.03 ± 0.03	0.03 ± 0.05	0.264
Reoperation (mean ± SD)	0.04 ± 0.03	0.04 ± 0.03	0.04 ± 0.03	0.917
*Preoperative labs and tests*				
C-reactive protein, mg/L (mean ± SD)	13.03 ± 31.35	13.21 ± 32.25	12.85 ± 30.46	0.846
White blood cell, × 10^3^/μL (mean ± SD)	7.88 ± 3.09	7.90 ± 2.94	7.86 ± 3.23	0.848
Hemoglobin, g/dl (mean ± SD)	13.03 ± 1.92	13.03 ± 1.95	13.03 ± 1.90	0.988
Hematocrit, % (mean ± SD)	38.22 ± 5.20	38.13 ± 5.20	38.32 ± 5.20	0.54
Platelet count, × 10^^3^/μL (mean ± SD)	221.16 ± 69.00	221.58 ± 67.25	220.74 ± 70.71	0.836
Creatinine, mg/dl (mean ± SD)	1.20 ± 1.06	1.21 ± 1.21	1.20 ± 0.89	0.889
Total bilirubin, mg/dl (mean ± SD)	0.73 ± 0.56	0.71 ± 0.56	0.75 ± 0.56	0.295
Prothrombin time—INR (mean ± SD)	1.02 ± 0.14	1.02 ± 0.16	1.02 ± 0.12	0.834
Preoperative positive rectal swabs (mean ± SD)	1.55 ± 1.64	0.77 ± 1.11	2.34 ± 1.70	< *0.001*
Preoperative positive nasal swab for MSSA/MRSA	1.70 ± 0.56	1.40 ± 0.57	1.99 ± 0.38	< *0.001*
*Home and preoperative medications*				
Diabetes medication				0.05
None, n (%)	878 (76.6)	439 (76.6)	439 (76.6)	
Diet only, n (%)	9 (0.8)	2 (0.3)	7 (1.2)	
Oral, n (%)	145 (12.7)	65 (11.3)	80 (14.0)	
Insulin, n (%)	114 (10.0)	67 (11.7)	47 (8.2)	
Bronchodilators, n (%)	56 (4.9)	29 (5.1)	27 (4.7)	0.891
Immunosuppressive therapy, n (%)	10 (0.9)	3 (0.5)	7 (1.2)	0.341
Angiotensin-converting enzyme inhibitors, n (%)	439 (38.3)	224 (39.1)	215 (37.5)	0.627
Antiplatelet agent, n (%)	113 (9.9)	62 (10.8)	51 (8.9)	0.322
Amiodarone, n (%)	40 (3.5)	19 (3.3)	21 (3.7)	0.872
Beta blockers, n (%)	673 (59)	334 (58.3)	339 (59.2)	0.81
Calcium channel blockers, n (%)	167 (14.6)	78 (13.6)	89 (15.5)	0.402
Inotropes, n (%)	9 (0.8)	2 (0.3)	7 (1.2)	0.181
Lipid-lowering therapy, n (%)	500 (43.6)	238 (41.5)	262 (45.7)	0.171
Preoperative anticoagulant therapy, n (%)	5 (0.5)	1 (0.2)	4 (0.7)	0.37
Preoperative cefazolin treatment, n (%)	1129 (98.5)	564 (98.4)	565 (98.6)	1
Preoperative mupirocin treatment, n (%)	918 (80.1)	463 (80.8)	455 (79.4)	0.604
*Procedure parameters*				
Length of stay in hospital, days, median (IQR)	11 (6)	11 (5)	11 (6)	0.78
Intra-aortic balloon pump, n (%)	37 (3.3)	16 (2.8)	21 (3.7)	0.504
Cardiopulmonary bypass time, minutes (mean ± SD)	105 ± 47	106 ± 47	104 ± 48	0.424
ICU length of stay postoperative, hours (mean ± SD)	110 ± 281	98 ± 247	124 ± 311	0.114
Time of mechanical ventilation, hours (mean ± SD)	54 ± 268	53 ± 307	54 ± 224	0.933
Left ventricular assist device implantation, n (%)	7 (0.6)	5 (0.9)	2 (0.3)	0.448
Blood transfusion postoperative, n (%)	293 (25.6)	149 (26.0)	144 (25.1)	0.786
Blood units administered during surgery, n (mean ± SD)	0.8 ± 1.2	0.8 ± 1.3	0.8 ± 1.2	0.188

BMI, body mass index; PCI, percutaneous coronary intervention; DES, drug-eluting stent; MSSA, methicillin-sensitive Staphylococcus aureus; MRSA, methicillin-resistant Staphylococcus aureus; ICU, intensive care unit.

No differences were observed in preoperative standard biochemical markers, including CRP, WBC count and other hemogram parameters, renal function, and coagulation. Interestingly, the Screen+ group showed a significantly higher number of positive rectal swabs and nasal swabs for MSSA/MRSA.

**Figure 3:**
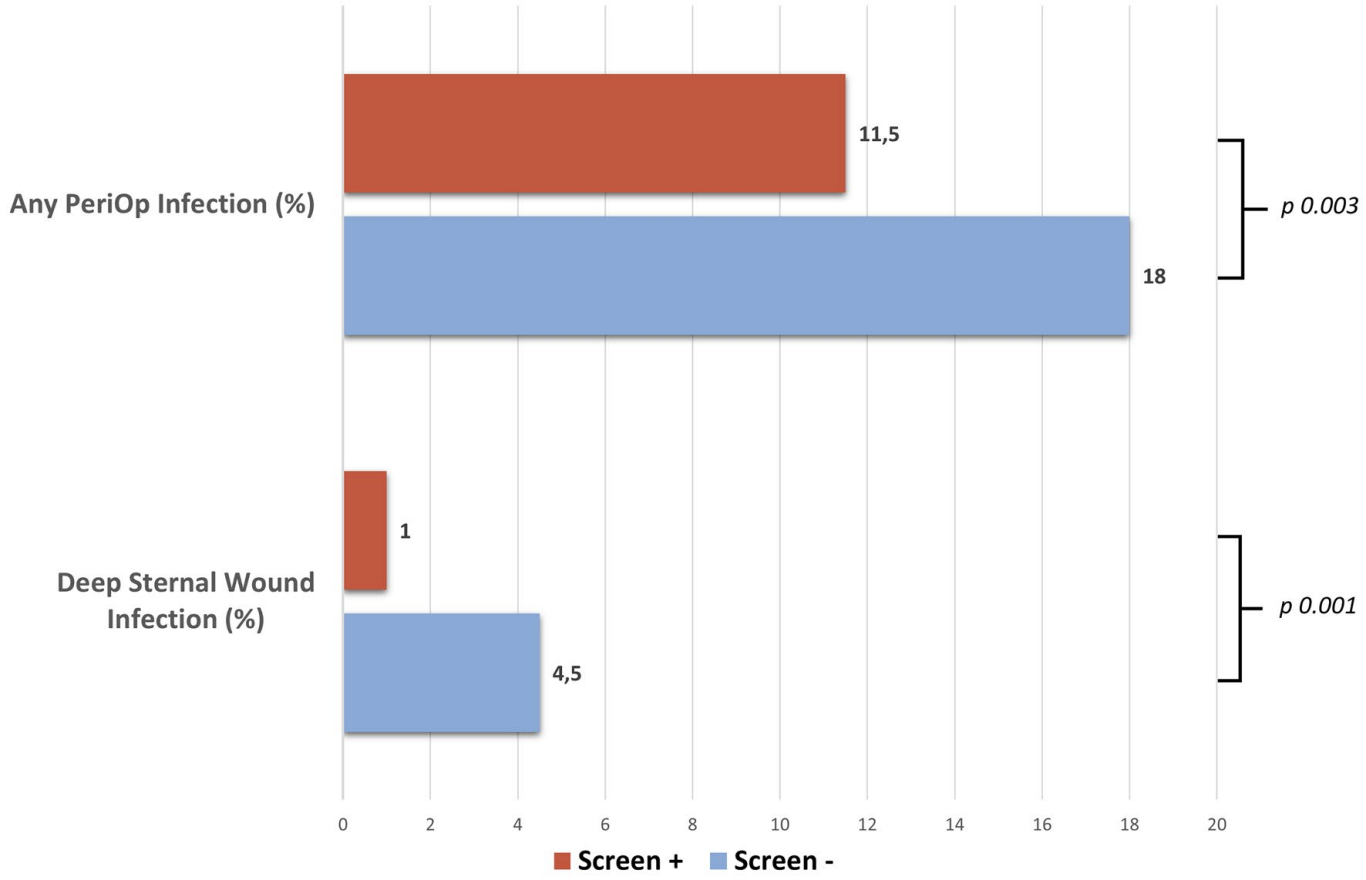
Study outcomes.

Despite the absence of significant differences in the predicted risk scores between the two groups regarding deep SWI (as well as morbidity or mortality, renal failure, and reoperation), the Screen+ group showed a significant reduced prevalence of perioperative infections compared with the Screen− group, including deep SWI (Table 2, Fig. 3). No differences were observed regarding short term (30 days) all causes death (Table 2).

**Table 2:** Study outcome comparison between Screen+ and Screen− groups.

Study outcomes	All	Screen−	Screen+	*p*-value
Any perioperative infection, n (%)	169 (14.8)	103 (18.0)	66 (11.5)	*0.003*
Deep sternal wound infection, n (%)	32 (2.8)	26 (4.5)	6 (1.0)	*0.001*
Short-term (30 days) all-cause mortality, n (%)	22 (1.9)	10 (1.8)	12 (2.1)	0.667

Significant values are in italics.

The analysis of A1c in the Screen+ group allowed the unmasking of 11.7% undiagnosed DM (negative history of DM with A1c ≥ 6.5%–48 mmol/mol) and a 22.9% dysglycemia due to prediabetes status (negative history with A1c between 6 and 6.5%—42 and 48 mmol/mol).

## Discussion

The primary objective of our study was to evaluate the impact of systematic diabetes screening and management on perioperative infectious outcomes and mortality among patients undergoing cardiac surgery.

Accordingly, our study has demonstrated for the first time that routine A1c evaluation at admission for patients undergoing cardiac surgery followed by DM specialist consult is associated with a significant reduction in perioperative infections at any location, including SWI.

The propensity score-matched procedure allowed the evaluation of differences between patients who participated in the screening program (Screen+) and those who did not (Screen−) by reducing the influence of the most common confounding factors, including pre-existing pathologies and parameters linked to a greater risk of infection.

Among the laboratory tests evaluated preoperatively, all routine parameters, including inflammatory markers, showed no differences between the two groups. Curiously, in the Screen+ group, a significantly greater number of patients had positive rectal and nasal swabs. These results can be partially explained by the increased exposure witnessed in recent years in our area. Furthermore, some nasal swab screening protocols have been modified over time at our facility. For example, since 2021 the nasal swab for detection of MSSA/MRSA has been included in an automatic order set applied to all patients admitted to cardiac surgery. This observation could possibly strengthen our result, since despite the greater positivity of the rectal and nasal swabs, the infectious outcomes were lower in the Screen+ group.

Despite the observed reduction in perioperative infections, our study did not demonstrate a statistically significant reduction in all-causes mortality within 30 days from discharge. Studies on the association between A1c and mortality have inconsistent results in the literature, in some cases finding an association with higher mortality, while in others not^23–26^. While our result was not aligned with our initial hypothesis, it underscores the multifactorial nature of mortality in the context of cardiac surgery, which may be influenced by factors beyond a history of diabetes or A1c value alone. Perioperative glycemic control, rather than A1c, is likely to be a greater contributor to mortality risk in cardiac surgery patients. Van Den Boom et al., in a 2018 study, found no association between A1c and 30-day mortality in a cohort of 6393 cardiac surgery patients; instead, they found a U-shaped curve in which extreme mean glycemic values (therefore, related to hypo- and hyperglycemic acute complications) are associated with a greater risk of mortality^27^.

The importance of A1c levels monitoring in patients referred to surgery (or not) is not new. Umpierrez et al., already in 2014, proposed the evaluation of A1c upon admission for both surgical and non-surgical diabetic patients to better manage glycemic control during hospitalization and optimize therapy upon discharge, but without an analysis of surgical outcomes^16^. More recently, Yong et al. demonstrated, in a large cohort of both diabetic and non-diabetic surgical patients, of which 574 underwent cardiac surgery, a clear association between A1c and outcomes such as major complications, ICU admission, and increased hospital length of stay (LOS)^7^. However, ours is the largest study to assess the impact of the implementation of glycemic management based on a comprehensive assessment of A1c on infectious disease outcomes.

Consistently, most research groups as well as international guidelines agree on the importance of an initial assessment of A1c as a necessary first step in setting up diagnostic and therapeutic strategies in the hospital setting, involving a DM specialist. As an additional benefit, A1c assessment, done systematically, allows for the identification of patients with undiagnosed DM, as we have observed in our study. Indeed, several reports have shown that undiagnosed DM in the hospital setting has an important role in the risk stratification of these patients compared with non-diabetic patients^28,29^. In our population, 24% of patients had a history of DM. The A1c analysis in the Screen+ group allowed a DM diagnosis in 67 more patients without known glucometabolic issues (12%), raising the rate of DM in the reference period to 36%, in line with literature data^17,30,31^.

Finally, the identification and treatment of diabetes at hospital admission is important from a healthcare and social point of view: an occasional diagnosis of diabetes reveals a complex disease that must be studied and treated, also to reduce the cardiovascular burden and avoid any rehospitalizations and reoperations in patients who are already severely compromised. If diabetes is already known, hospitalization may be an opportunity to assess the efficacy of the therapeutic regimen, even considering the availability of new antidiabetic medications with cardioprotective effects.

Despite novelty and merits our study has limitations that need to be discussed. First, even if propensity score matching was used to reduce any confounding factors, it is not possible to rule out potential selection biases, due to unobserved variables for example and to the retrospective nature of the study itself. However, the STS database our data are extracted for is well known for its completeness and thoroughness in terms of variables collection, so we are confident that the most important confounders have been nulled by the matching procedure. In addition, we recognize that the A1c dosage may be affected by any recent transfusions and other conditions such as hemoglobinopathies, anemia and iron deficiency. However, having considered the type of operation, urgency, other comorbidities, and some parameters of interest, such as hemoglobin level, in the propensity score, there should be limited potential bias in this regard. Furthermore, the choice of A1c evaluation for the diagnosis of unknown diabetes is supported by recent literature where, in some settings, the replacement of the oral glucose tolerance test (OGTT) with A1c-based diagnosis appears justified^32^. Despite potential temporal bias, the surgical methods across the study periods were consistent, with no novel surgery techniques introduced and exclusion of patients undergoing minimally invasive or percutaneous procedures. This consistency, along with propensity matching that included reasons for admission, priority of intervention and surgical details, helps mitigate the impact of any "surgical era effect" on the study's outcomes. Another issue is that the study results cannot necessarily be applied to all healthcare settings or regions as local factors and procedures can vary widely; UPMC | IRCCS ISMETT operates in a region endemic for multidrug-resistant organisms which may have influenced the study results. Regarding the study outcomes, one limitation is the lack of more specific data on the type, site, and severity of infection. However, we have focused our analysis on SWI, which is a well-established marker of infective outcome in patients referred to cardiac surgery. While the study did not find a statistically significant reduction in 30-day mortality, it is important to note that this timeframe may not capture longer-term impacts, and the study follow-up may be too short to assess the full range of mortality outcomes. Finally, another limitation of the study is the lack of specific data about the cost differences associated with higher infectious complications. However, cost analysis on this specific study might not be entirely applicable since the analysis on groups over different time periods and the matching on prophylactic and surgical parameters could introduce a significant bias regarding the weight of medical and non-medical direct costs.

## Conclusion

A1c control for all patients undergoing cardiac surgery in our facility has led to better pre-surgical risk stratification, reduction of infectious complications, and the identification of a significant number of previously undiagnosed cases of diabetes. Our results suggest that a proactive approach to glycemic control may contribute to diminishing the risk of infectious complications in the perioperative period.

In diabetic patients identified upon admission to the hospital with non-target A1c levels or in those with undiagnosed diabetes, prompt management by a specialist diabetes team coordinating intensive treatment can reduce the infectious risks associated with surgery. Additionally, identifying patients with undiagnosed diabetes and initiating proper phenotyping by a specialist can reduce acute diabetes-related complications in the perioperative period, such as severe hypoglycemia and hyperglycemia.

The positive results obtained with this novel process at our facility lay the foundations for investing resources and personnel in the more accurate management of DM in hospitals, performed by specialized diabetes teams, encouraging the use of appropriate technologies (e.g., real-time continuous glucose monitoring (CGM) and insulin pump) with the aim of reducing postoperative infectious complications. Furthermore, the approach poses specific outcomes that have a positive impact on healthcare costs, which should be carefully evaluated with further studies.

### Supplementary Information

Supplementary Information.

## Data Availability

The datasets generated and/or analysed during the current study are not publicly available due to institutional internal policies but could be available from the corresponding author on reasonable request following the necessary assessments and agreements.

## Abbreviations

A1C: Glycated hemoglobin
BMI: Body mass index
CABG: Coronary artery bypass graft
CDC: Centers for disease control and prevention
CGM: Continuous glucose monitoring
COPD: Chronic obstructive pulmonary disease
CRP: C-reactive protein
DES: Drug-eluting stent
DM: Diabetes mellitus
ESBL: Extended-spectrum beta-lactamase
IRB: Institutional review board
LOS: Length of stay
MDRO: Multidrug-resistant organism
MELD: Model for end-stage liver disease
MRSA: Methicillin-resistant staphylococcus aureus
MSSA: Methicillin-sensitive staphylococcus aureus
NHSN: National healthcare safety network
NYHA: New york heart association
OGTT: Oral glucose tolerance test
PCI: Percutaneous coronary intervention
SD: Standard deviation
STS: Society of thoracic surgeons
SWI: Surgical wound infection
VRE: Vancomycin-resistant enterococci
WBC: White blood cell
