# Supplementary material for: Impact of systematic diabetes screening on peri-operative infections in patients undergoing cardiac surgery
Source: Sci Rep. 2024 Jun 20;14:14182. doi: 10.1038/s41598-024-65064-7 (PMC11187113; doi:10.1038/s41598-024-65064-7)
Supplement: Supplementary file 1 — Supplementary Information. [file 41598_2024_65064_MOESM1_ESM.docx]

**SUPPLEMENTARY INFORMATION
Impact of Systematic Diabetes Screening on Peri-Operative Infections in Patients Undergoing Cardiac Surgery
A Mattina et al.**

| **Table S1.** Whole pre-matching population characteristics | |
| --- | --- |
|  | **All** |
| n. | 4526 |
| **Characteristics and medical history** |  |
| Age, years (mean ± DS) | 64.6 ± 12.3 |
| Females, n(%) | 1597 (35.2) |
| Height, cm (mean ± DS) | 165 ± 9.5 |
| Weight, Kg (mean ± DS) | 74.9 ± 14.6 |
| BMI, kg/m² (mean ± DS) | 27.4 ± 4.6 |
| Smoke, n(%) | 734 (16.2) |
| Hemo-dyalisis, n(%) | 87 (1.9) |
| Liver disease, n(%) | 147 (3.2) |
| Diabetes mellitus, n (%) | 1101 (24.3) |
| History of infective endocarditis, n(%) | 161 (3.5) |
| History of cancer, n(%) | 216 (4.8) |
| **Cardiovascular history** |  |
| Arterial hypertension, n(%) | 3538 (78.0) |
| Coronary artery disease, n(%) | 958 (21.1) |
| Peripheral arterial disease, n(%) | 165 (3.6) |
| Carotid artery stenting, n(%) | 263 (5.8) |
| Cerebrovascular accident, n(%) | 542 (11.9) |
| Acute myocardial infarct, n(%) | 689 (15.2) |
| Acute heart failure, n(%) | 815 (18.0) |
| NYHA Class III/IV, n(%) | 722 (16) |
| Arrhythmia, n(%) | 1066 (23.5) |
| Pacemaker implantation, n(%) | 43 (0.9) |
| Left ventricular assist device, n(%) | 23 (0.5) |
| History of PCI and DES implantation, n(%) | 545 (12.0) |
| **STS predicted risk scores** |  |
| Morbidity or mortality (mean ± DS) | 0.02 ± 0.02 |
| Deep sternal wound infection (mean ± DS) | 0.00 ± 0.00 |
| Renal failure (mean ± DS) | 0.03 ± 0.04 |
| Reoperation (mean ± DS) | 0.04 ± 0.03 |
| **Preoperative labs and tests** |  |
| C-reactive protein, mg/L (mean ± DS) | 12.03 ± 30.7 |
| White blood cell, x 10^³/μL (mean ± DS) | 7.95 ± 2.87 |
| Hemoglobin, g/dl (mean ± DS) | 13.05 ± 1.93 |
| Hematocrit, % (mean ± DS) | 38.46 ± 5.16 |
| Platelet count, x 10^³/μL (mean ± DS) | 219.06 ± 68.46 |
| Creatinine, mg/dl (mean ± DS) | 1.24 ± 1.08 |
| Total bilirubin, mg/dl (mean ± DS) | 0.75 ± 0.60 |
| Prothrombin time - INR (mean ± DS) | 1.05 ± 0.28 |
| Preoperative positive rectal swabs (mean ± DS) | 0.99 ± 1.49 |
| Preoperative positive nasal swab for MSSA/MRSA | 1.70 ± 0.56 |
| **Home and preoperative medications** |  |
| *Diabetes medication* |  |
| *None, n(%)* | 3494 (77.0) |
| *Diet only, n(%)* | 47 (1.0) |
| *Oral, n(%)* | 537 (11.8) |
| *Insulin, n(%)* | 458 (10.1) |
| Bronchodilators, n(%) | 158 (3.5) |
| Immunosuppressive therapy, n(%) | 41 (0.9) |
| Angiotensin-converting enzyme inhibitors, n(%) | 1744 (38.4) |
| Antiplatelet agent, n(%) | 581 (12.8) |
| Amiodarone, n(%) | 220 (4.8) |
| Beta blockers, n(%) | 2562 (56.5) |
| Calcium channel blockers, n(%) | 630 (13.9) |
| Inotropes, n(%) | 33 (0.7) |
| Lipid-lowering therapy, n(%) | 1889 (41.6) |
| Preoperative anticoagulant therapy, n(%) | 36 (0.8) |
| Preoperative cafzolin treatment, n(%) | 4279 (94.3) |
| Preoperative mupirocin treatment, n(%) | 2132 (47.0) |
| **Procedure parameters** |  |
| Length of stay in hospital, days, median (IQR) | 10 (6) |
| Intra-aortic balloon pump, n(%) | 160 (3.5) |
| Cardiopulmonary bypass time, minutes (mean ± DS) | 107.58 ± 50.07 |
| ICU length of stay postoperative, hours (mean ± DS) | 108.47 ± 285.60 |
| Time of mechanical ventilation, hours (mean ± DS) | 49.19 ± 235.30 |
| Left ventricular assist device implantation, n(%) | 61 (1.3) |
| Blood transfusion postoperative, n(%) | 1272 (28.0) |
| Blood units administered during surgery, n (mean ± DS) | 0.87 ± 1.33 |
|  |  |
| BMI: body mass index. PCI: percutaneous coronary intervention. DES: drug-eluting stent. MSSA: Methicillin-sensitive Staphylococcus aureus. MRSA: methicillin-resistant Staphylococcus aureus. ICU: intensive care unit. | |

| **Table S2.** Matching variables distribution between groups (pre-match assessment) | | | |
| --- | --- | --- | --- |
|  | **Screen -** | **Screen +** | **SMD** |
| n. | 3929 | 597 |  |
| Age, years (mean ± SD) | 64.10 ± 12.24 | 64.74 ± 11.57 | 0.054 |
| Females, n (%) | 1367 (34.8) | 198 (33.2) | 0.034 |
| BMI, kg/m² (mean ± SD) | 27.40 ± 4.56 | 27.26 ± 4.60 | 0.031 |
| **Admission reason** |  |  | 0.154 |
| *Aortic or mitral valve disease, n (%)* | 1272 (32.4) | 212 (35.5) |  |
| *Native coronary atherosclerosis, non-ST-elevation MI, n (%)* | 698 (17.8) | 94 (15.7) |  |
| *Thoracic aneurysm (without rupture), n (%)* | 193 (4.9) | 30 (5.0) |  |
| *Acute and subacute forms of ischemic heart disease, n (%)* | 127 (3.2) | 16 (2.7) |  |
| *Thoracoabdominal aorta dissection, n (%)* | 46 (1.2) | 2 (0.3) |  |
| *Acute decompensated heart failure, n (%)* | 71 (1.8) | 8 (1.3) |  |
| *Mixed complications from heart valve prosthesis, n (%)* | 56 (1.4) | 4 (0.7) |  |
| *Obstructive hypertrophic cardiomyopathy, n (%)* | 46 (1.2) | 9 (1.5) |  |
| *Other (any category not included among those above), n (%)* | 1420 (36.1) | 222 (37.2) |  |
| **Operation priority** |  |  | 0.443 |
| *Elective, n (%)* | 3417 (87.0) | 468 (78.4) |  |
| *Urgent, n (%)* | 338 (8.6) | 127 (21.3) |  |
| *Emergent, n (%)* | 160 (4.1) | 2 (0.3) |  |
| *Emergent salvage, n (%)* | 14 (0.4) | 0 (0.0) |  |
| **Incidence** |  |  | 0.124 |
| *First cardiovascular surgery, n (%)* | 3558 (90.6) | 554 (92.8) |  |
| *First re-op cardiovascular surgery, n (%)* | 304 (7.7) | 39 (6.5) |  |
| *Second re-op cardiovascular surgery, n (%)* | 49 (1.2) | 4 (0.7) |  |
| *Third re-op cardiovascular surgery, n (%)* | 16 (0.4) | 0 (0.0) |  |
| *Fourth or more re-op cardiovascular surgery, n (%)* | 2 (0.1) | 0 (0.0) |  |
| **Medical history and risk scores** |  |  |  |
| Smoking, n (%) | 634 (16.1) | 108 (18.1) | 0.052 |
| Hemodialysis, n (%) | 74 (1.9) | 11 (1.8) | 0.003 |
| Liver disease, n (%) | 131 (3.3) | 16 (2.7) | 0.038 |
| MELD risk score (mean ± SD) | 9.06 ± 3.52 | 8.37 ± 2.83 | 0.217 |
| Predicted risk of mortality (mean ± SD) | 0.02 ± 0.02 | 0.02 ± 0.03 | 0.024 |
| History of infective endocarditis, n (%) | 147 (3.7) | 17 (2.8) | 0.05 |
| Infective endocarditis at admission time, n (%) | 96 (2.4) | 9 (1.5) | 0.067 |
| Chronic obstructive pulmonary disease |  |  | 0.121 |
| *No, n (%)* | 3401 (86.6) | 507 (84.9) |  |
| *Mild, n (%)* | 33 (0.8) | 7 (1.2) |  |
| *Moderate, n (%)* | 474 (12.1) | 83 (13.9) |  |
| *Severe, n (%)* | 21 (0.5) | 0 (0.0) |  |
| **Cardiovascular history** |  |  |  |
| Arterial hypertension, n (%) | 3038 (77.3) | 481 (80.6) | 0.08 |
| Coronary artery disease, n (%) | 930 (23.7) | 42 (7.0) | 0.474 |
| Peripheral artery disease, n (%) | 131 (3.3) | 31 (5.2) | 0.092 |
| Cerebrovascular accident, n (%) | 480 (12.2) | 60 (10.1) | 0.069 |
| Coronary artery bypass graft surgery, n (%) | 60 (1.5) | 4 (0.7) | 0.082 |
| Percutaneous coronary intervention, n (%) | 541 (13.8) | 70 (11.7) | 0.061 |
| Acute myocardial infarction, n (%) | 618 (15.7) | 69 (11.6) | 0.122 |
| Acute heart failure, n (%) | 812 (20.7) | 32 (5.4) | 0.467 |
| Heart failure |  |  | 0.666 |
| *Not documented, n (%)* | 1479 (37.6) | 107 (17.9) |  |
| *NYHA Class I, n (%)* | 27 (0.7) | 15 (2.5) |  |
| *NYHA Class II, n (%)* | 1856 (47.2) | 321 (53.8) |  |
| *NYHA Class III, n (%)* | 318 (8.1) | 146 (24.5) |  |
| *NYHA Class IV, n (%)* | 249 (6.3) | 8 (1.3) |  |
| Preoperative LVEF, % (mean ± SD) | 56.35 ± 10.85 | 56.07 ± 10.38 | 0.027 |
| **Preoperative laboratory parameters** |  |  |  |
| C-reactive protein, mg/L (mean ± SD) | 11.91 ± 30.90 | 13.22 ± 30.85 | 0.043 |
| White blood cell, x 10^³/μL (mean ± SD) | 7.97 ± 2.82 | 7.86 ± 3.19 | 0.035 |
| Hemoglobin, g/dl (mean ± SD) | 13.08 ± 1.94 | 13.00 ± 1.91 | 0.043 |
| Creatinine, mg/dl (mean ± SD) | 1.24 ± 1.10 | 1.21 ± 0.91 | 0.037 |
| **Prophylactic therapy** |  |  |  |
| Preoperative cefazolin treatment, n (%) | 3675 (93.5) | 589 (98.7) | 0.267 |
| Preoperative mupirocin treatment, n (%) | 1671 (42.5) | 478 (80.1) | 0.835 |
| **Procedure parameters** |  |  |  |
| Intra-aortic balloon pump, n (%) | 135 (3.4) | 21 (3.5) | 0.004 |
| Cardiopulmonary bypass time, minutes (mean ± SD) | 108.14 ± 51.13 | 103.44 ± 47.34 | 0.095 |
| Blood units administered during surgery, n (mean ± SD) | 0.85 ± 1.35 | 0.85 ± 1.18 | 0.005 |
| ICU length of stay postoperative, hours (mean ± SD) | 84.45 ± 197.78 | 78.20 ± 114.59 | 0.039 |
| Time of mechanical ventilation, hours (mean ± SD) | 47.82 ± 236.66 | 52.54 ± 219.10 | 0.021 |
|  |  |  |  |
| SMD: standardized mean difference (measure of distance between two group means regarding one or more variables. Here it is used as a balance measure of individual covariates before and after propensity score matching). BMI: body mass index. MELD: model for end-stage liver disease. NYHA: New York Heart Association. ICU: intensive care unit. | | | |

| **Table S3.** Matching variable distribution between groups (post-match assessment) | | | |
| --- | --- | --- | --- |
|  | **Screen -** | **Screen +** | **SMD** |
| n. | 573 | 573 |  |
| Age, years (mean ± SD) | 64.69 ± 11.55 | 64.78 ± 11.60 | 0,007 |
| Females, n (%) | 187 (32.6) | 190 (33.2) | 0,011 |
| BMI, kg/m² (mean ± SD) | 27.30 ± 4.69 | 27.27 ± 4.57 | 0,005 |
| **Admission reason** |  |  | 0,088 |
| *Aortic or mitral valve disease, n (%)* | 193 (33.7) | 203 (35.4) |  |
| *Native coronary atherosclerosis, non-ST-elevation MI, n (%)* | 97 (16.9) | 89 (15.5) |  |
| *Thoracic aneurysm (without rupture), n (%)* | 29 (5.1) | 29 (5.1) |  |
| *Acute and subacute forms of ischemic heart disease, n (%)* | 18 (3.1) | 16 (2.8) |  |
| *Thoracoabdominal aorta dissection, n (%)* | 1 (0.2) | 2 (0.3) |  |
| *Acute decompensated heart failure, n (%)* | 5 (0.9) | 7 (1.2) |  |
| *Mixed complications from heart valve prosthesis, n (%)* | 7 (1.2) | 4 (0.7) |  |
| *Obstructive hypertrophic cardiomyopathy, n (%)* | 9 (1.6) | 9 (1.6) |  |
| *Other (any category not included among those above), n (%)* | 214 (37.3) | 214 (37.3) |  |
| **Operation priority** |  |  | 0,032 |
| *Elective, n (%)* | 451 (78.7) | 456 (79.6) |  |
| *Urgent, n (%)* | 119 (20.8) | 115 (20.1) |  |
| *Emergent, n (%)* | 3 (0.5) | 2 (0.3) |  |
| *Emergent salvage, n (%)* | 0 (0.0) | 0 (0.0) |  |
| **Incidence** |  |  | <0.001 |
| *First cardiovascular surgery, n (%)* | 530 (92.5) | 530 (92.5) |  |
| *First re-op cardiovascular surgery, n (%)* | 39 (6.8) | 39 (6.8) |  |
| *Second re-op cardiovascular surgery, n (%)* | 4 (0.7) | 4 (0.7) |  |
| *Third re-op cardiovascular surgery, n (%)* | 0 (0.0) | 0 (0.0) |  |
| *Fourth or more re-op cardiovascular surgery, n (%)* | 0 (0.0) | 0 (0.0) |  |
| **Medical history and risk scores** |  |  |  |
| Smoking, n (%) | 93 (16.2) | 102 (17.8) | 0,042 |
| Hemodialysis, n (%) | 11 (1.9) | 10 (1.7) | 0,013 |
| Liver disease, n (%) | 17 (3.0) | 16 (2.8) | 0,01 |
| MELD risk score (mean ± SD) | 8.53 ± 2.72 | 8.39 ± 2.83 | 0,049 |
| Predicted risk of mortality (mean ± SD) | 0.02 ± 0.03 | 0.02 ± 0.03 | 0,015 |
| History of infective endocarditis, n (%) | 11 (1.9) | 8 (1.4) | 0,041 |
| Infective endocarditis at admission time, n (%) | 22 (3.8) | 16 (2.8) | 0,059 |
| Chronic obstructive pulmonary disease |  |  | 0,044 |
| *No, n (%)* | 478 (83.4) | 487 (85.0) |  |
| *Mild, n (%)* | 6 (1.0) | 6 (1.0) |  |
| *Moderate, n (%)* | 89 (15.5) | 80 (14.0) |  |
| *Severe, n (%)* | 0 (0.0) | 0 (0.0) |  |
| **Cardiovascular history** |  |  |  |
| Arterial hypertension, n (%) | 456 (79.6) | 460 (80.3) | 0,017 |
| Coronary artery disease, n (%) | 32 (5.6) | 42 (7.3) | 0,071 |
| Peripheral artery disease, n (%) | 19 (3.3) | 28 (4.9) | 0,079 |
| Cerebrovascular accident, n (%) | 55 (9.6) | 57 (9.9) | 0,012 |
| Coronary artery bypass graft surgery, n (%) | 4 (0.7) | 4 (0.7) | <0.001 |
| Percutaneous coronary intervention, n (%) | 59 (10.3) | 67 (11.7) | 0,045 |
| Acute myocardial infarction, n (%) | 63 (11.0) | 68 (11.9) | 0,027 |
| Acute heart failure, n (%) | 25 (4.4) | 30 (5.2) | 0,041 |
| Heart failure |  |  | 0,061 |
| *Not documented, n (%)* | 110 (19.2) | 107 (18.7) |  |
| *NYHA Class I, n (%)* | 10 (1.7) | 11 (1.9) |  |
| *NYHA Class II, n (%)* | 331 (57.8) | 320 (55.8) |  |
| *NYHA Class III, n (%)* | 116 (20.2) | 127 (22.2) |  |
| *NYHA Class IV, n (%)* | 6 (1.0) | 8 (1.4) |  |
| Preoperative LVEF, % (mean ± SD) | 116 | 56.18 ± 10.38 | 0,012 |
| **Preoperative laboratory parameters** |  |  |  |
| C-reactive protein, mg/L (mean ± SD) | 13.21 ± 32.25 | 12.85 ± 30.46 | 0,012 |
| White blood cell, x 10^³/μL (mean ± SD) | 7.90 ± 2.94 | 7.86 ± 3.23 | 0,011 |
| Hemoglobin, g/dl (mean ± SD) | 13.03 ± 1.95 | 13.03 ± 1.90 | 0,001 |
| Creatinine, mg/dl (mean ± SD) | 1.21 ± 1.21 | 1.20 ± 0.89 | 0,008 |
| **Prophylactic therapy** |  |  |  |
| Preoperative cefazolin treatment, n (%) | 564 (98.4) | 565 (98.6) | 0,014 |
| Preoperative mupirocin treatment, n (%) | 463 (80.8) | 455 (79.4) | 0,035 |
| **Procedure parameters** |  |  |  |
| Intra-aortic balloon pump, n (%) | 16 (2.8) | 21 (3.7) | 0,049 |
| Cardiopulmonary bypass time, minutes (mean ± SD) | 106.26 ± 46.46 | 104.04 ± 47.63 | 0,047 |
| Blood units administered during surgery, n (mean ± SD) | 0.75 ± 1.28 | 0.84 ± 1.18 | 0,078 |
| ICU length of stay postoperative, hours (mean ± SD) | 78.87 ± 146.94 | 78.82 ± 116.72 | <0.001 |
| Time of mechanical ventilation, hours (mean ± SD) | 52.95 ± 307.18 | 54.29 ± 223.46 | 0,005 |
|  |  |  |  |
| SMD: standardized mean difference (measure of distance between two group means regarding one or more variables. Here it is used as a balance measure of individual covariates before and after propensity score matching). BMI: body mass index. MELD: model for end-stage liver disease. NYHA: New York Heart Association. ICU: intensive care unit. | | | |

| **Table S4.** Study outcome comparison between the Screen+ and Screen- groups for patients referred to heart tranplantation (HTx) and left ventricular assist device (LVAD) implantation. | | | | |
| --- | --- | --- | --- | --- |
| **Study outcomes for HTx patients** | **All (N14)** | **Screen -** | **Screen +** | **p-value** |
| Any perioperative infection, n(%) | 9 (64.3) | 2 (50) | 7 (70) | 0.48 |
| Deep sternal wound infection, n(%) | 1 (7.1) | 1 (25) | 0 (0) | 0.101 |
| Short term (30 days) all causes death, n(%) | 3 (21.4) | 1 (25) | 2 (20) | 0.837 |
| **Study outcomes for LVAD implant patients** | **All (N13)** | **Screen -** | **Screen +** | **p-value** |
| Any perioperative infection, n(%) | 8 (61.5) | 3 (60) | 4 (30.8) | 0.48 |
| Deep sternal wound infection, n(%) | 0 (0) | 0 (0) | 0 (0) | - |
| Short term (30 days) all causes death, n(%) | 1 (7.7) | 0 (0) | 1 (12.5) | 0.411 |

**Figure S1. Admission Reasons**

**Figure S2. Distribution of Propensity Scores**


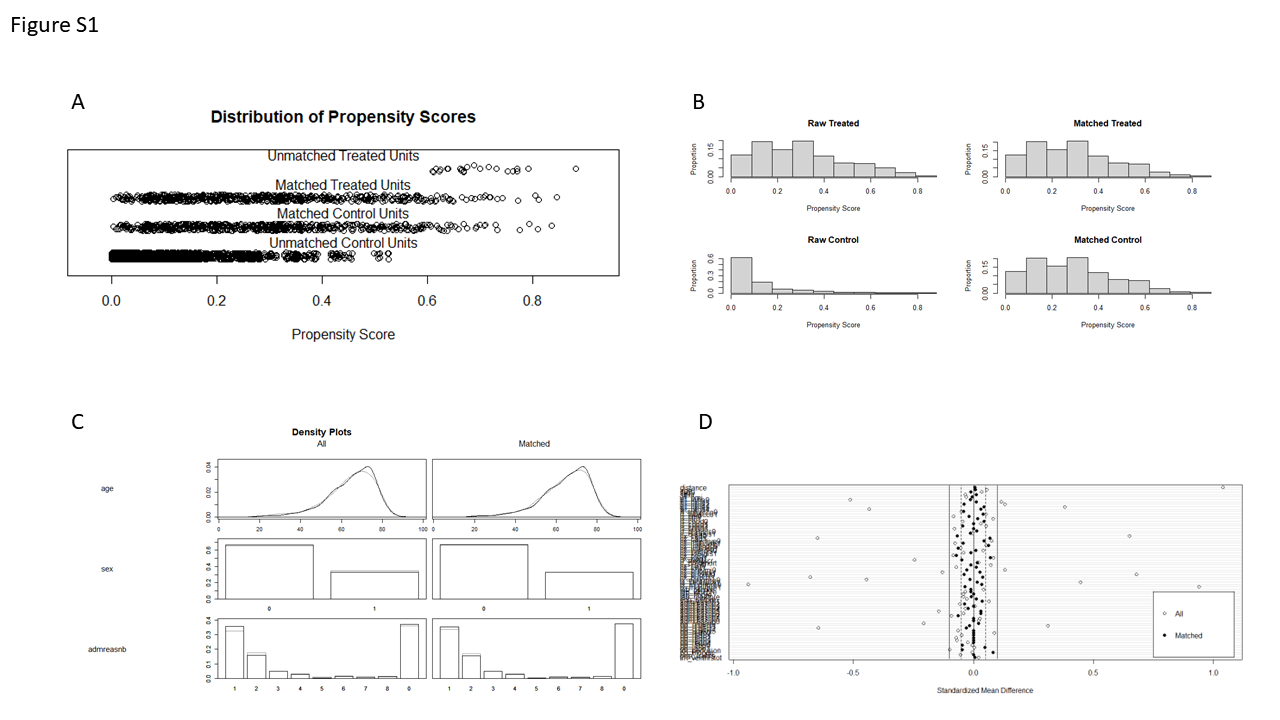


A: dot plot showing overall distribution of computed propensity scores stratified by matched and unmatched patients. B: histograms of propensity scores for raw (i.e. pre-match) and matched (i.e. post-matching) patients in the “treated” (i.e. referred to DOCS screening plan) or control/untreated (i.e. not referred to DOCS screening plan) groups. C: k-density plots of age, sex and admission reason (i.e. primary health issue for which the patient was referred to cardiac surgery) considering all patients together (on the left) or just matched pairs (on the right side). D: “love-plot” showing the standardized mean difference (SMD) for variables included in the propensity score building process, for the whole population and for matched pairs as well.

**Variable definitions from STS Registry**

List of the most common definitions present in the text adapted for a better interpretation of the data. For the complete list and more information refer to the STS National Database website (<https://www.sts.org/sts-national-database>).

- BMI - System calculated BMI. Tobacco Use - Indicate current (within 30 days prior to admission) or previous use of any tobacco product, including Cigarettes, Pipe, Cigars, Smokeless Cans, Other tobacco products (orbs, strips, sticks, hookah, etc.)
- Diabetes mellitus - History of diabetes diagnosed and/or treated by a healthcare provider. Hemoglobin A1c >=48 mmol/mol (6.5%) is indicative of diabetes.
- Dyslipidemia - Chronic renal dialysis - Indicate whether the patient is currently (prior to surgery) undergoing dialysis on a routine.
- Hypertension – Indicate if the patient has a current diagnosis of hypertension defined by any 1 of the following:
  - History of hypertension diagnosed and treated with medication, diet, and/or exercise
  - Currently undergoing pharmacological therapy for treatment of hypertension
- Endocarditis - Indicate whether the patient has a history of endocarditis. Endocarditis must meet the current CDC definition.
- Pneumonia - Indicate whether patient has a recent (within 30 days) or remote (more than 30 days) history of pneumonia.
- Liver Disease - Indicate whether the patient has a history of hepatitis B, hepatitis C, drug induced hepatitis, auto-immune hepatitis, cirrhosis, portal hypertension, esophageal varices, liver transplant, or congestive hepatopathy. Exclude NASH in the absence of cirrhosis.
- Cancer within 5 years - Indicate whether the patient has a history of cancer diagnosed within 5 years of procedure. Do not capture low grade skin cancers such as basal cell or squamous cell carcinoma.
- Peripheral Arterial Disease - Indicate whether the patient has a history of peripheral arterial disease (includes upper and lower extremity, renal, mesenteric, and abdominal aortic systems).
- Cerebrovascular Disease - Indicate whether the patient has a current or previous history of any of the following:
  - Stroke: Stroke is an acute episode of focal or global neurological dysfunction caused by brain, spinal cord, or retinal vascular injury as a result of hemorrhage or infarction, where the neurological dysfunction lasts for greater than 24 hours
  - TIA: is defined as a transient episode of focal neurological dysfunction caused by brain, spinal cord, or retinal ischemia, without acute infarction, where the neurological dysfunction resolves within 24 hours
  - Noninvasive or invasive arterial imaging test demonstrating >=50% stenosis of any of the major extracranial or intracranial vessels to the brain
  - Vertebral artery and internal carotid and intracranial consistent with atherosclerotic disease with document presence as CVD. External carotid disease is excluded
  - Previous cervical or cerebral artery revascularization surgery or percutaneous intervention
  - Brain/cerebral aneurysm
  - Occlusion of vertebral artery, internal carotid artery, and intracranial due to dissection

This does not include chronic (nonvascular) neurological diseases or other acute neurological insults such as metabolic and anoxic ischemic encephalopathy. Subdural hematoma or AVM is not cerebral vascular disease.

- Chronic lung Disease - Indicate whether the patient has chronic lung disease, and the severity level according to the following classification:
  - No
  - Mild: FEV1 60% to 75% of predicted or on chronic inhaled or oral bronchodilator therapy
  - Moderate: FEV1 50% to 59% of predicted or on chronic oral/systemic steroid therapy aimed at lung disease
  - Severe: FEV1 < 50% or Room Air pO2 < 60 or pCO2 > 50
  - CLD present, severity not documented
  - Unknown
- Inhaled medication - Indicate whether oral and/or inhaled bronchodilator or inhaled (not oral or IV) steroid medications were in use by the patient routinely prior to this procedure.
- Sleep apnea - Indicate whether patient has a diagnosis of sleep apnea (may be described as obstructive sleep apnea or OSA).
- Previous Cardiac Intervention - Indicate whether the patient has undergone any previous cardiovascular intervention, either surgical or non-surgical, which may include those done during the current admission.
- Previous MI - Indicate if the patient has had at least one documented previous myocardial infarction at any time prior to this surgery.
- Heart Failure –NYHA - Indicate whether there is physician documentation or report that the patient has a history of heart failure, Indicate the patient's worst dyspnea or functional class, coded as the New York Heart Association (NYHA) classification documented by a MD/Provider within the past 2 weeks.
- Intra-op blood products - Indicate whether blood products were transfused any time intraoperatively during the initial surgery. Intraoperatively is defined as any blood started after OR Entry before OR Exit.
- Cardiac Arrhythmia - Indicate whether the patient has a history of a cardiac rhythm disturbance prior to the induction of anesthesia.
- Cardiopulmonary bypass time - Indicate the total number of minutes that systemic return is diverted into the cardiopulmonary bypass (CPB) circuit and returned to the systemic system. This time period (Cardiopulmonary Bypass Time) includes all periods of cerebral perfusion and sucker bypass. This time period (Cardiopulmonary Bypass Time) excludes any circulatory arrest and modified ultrafiltration periods. If more than one period of CPB is required during the surgical procedure, the sum of all the CPB periods will equal the total number of CPB minutes.
- Operation priority
  - Elective‐ The patient's cardiac function has been stable in the days or weeks prior to the operation. The procedure could be deferred without increased risk of compromised cardiac outcome.
  - Urgent ‐ Procedure required during same hospitalization in order to minimize chance of further clinical deterioration.
  - Emergent ‐ Patients requiring emergency operations will have ongoing, refractory (difficult, complicated, and/or unmanageable) unrelenting cardiac compromise, with or without hemodynamic instability, and not responsive to any form of therapy except cardiac surgery. An emergency operation is one in which there should be no delay in providing operative intervention.
  - Emergent Salvage ‐ The patient is undergoing CPR en-route to the OR prior to anesthesia induction or has ongoing ECMO to maintain life. ECMO: ECMO is to be captured as a status of ‘Salvage’ in sequence 1975 and as ‘Resuscitation – Yes’ in sequence 935. ECMO is a supportive modality and not a procedural type. The risk of the patient on ECMO is accounted for when ‘Status = salvage’ and should be left in the intended procedural category.
- Chronic renal dialysis - Indicate whether the patient is currently (prior to surgery) undergoing dialysis on a routine.
- Intra-operative blood products received - Indicate whether blood products were transfused any time intraoperatively during the initial surgery. Intraoperatively is defined as any blood started after OR Entry before OR Exit.
- Glycated Hemoglobin, mmol/mol: Indicate the pre-operative HbA1c level closest to the date and time prior surgery but prior to anesthetic management (induction area or operating room).
- Last White Blood Cell count, x109/L: Indicate the pre-operative White Blood Cell (WBC) count closest to the date and time prior to surgery but prior to anesthetic management (induction area or operating room).
- Total bilirubin, mg/dL: Indicate the total Bilirubin closest to the date and time prior to surgery but prior to anesthetic management (induction area or operating room).
- Last hematocrit, %: Indicate the pre-operative Hematocrit level at the date and time closest to surgery but prior to anesthetic management (induction area or operating room). Capture only measured hematocrit levels, not calculated values.
- Platelets, x109/L: Indicate the platelet count closest to the date and time prior to surgery but prior to anesthetic management (induction area or operating room).
